# Amantadine-Induced Cardiac Arrest in a Patient With COVID-19

**DOI:** 10.7759/cureus.21345

**Published:** 2022-01-17

**Authors:** Bibek Bakhati, Victoria M Sibi, Armugam P Mekala, Joshua A Ronen, Sai S Mungara

**Affiliations:** 1 Internal Medicine, Texas Tech University Health Sciences Center Permian Basin, Odessa, USA; 2 Medicine, North-Western State Medical University named after I.I. Mechnikov, St. Petersburg, RUS; 3 Internal Medicine, University of California San Francisco Medical Center, San Francisco, USA; 4 Division of Hospital Medicine, University of California San Francisco School of Medicine, San Francisco, USA

**Keywords:** emergency, covid-19, cardiac arrest, acls, amantadine

## Abstract

Amantadine, which is known for its antiviral activity, is presently used as therapy for Parkinson's disease. Adverse effects, such as cardiac arrhythmias, have been described in patients after ingestion of amantadine. Here, we present a patient who suffered a cardiac arrest following ingestion of a low dose of amantadine.

A 71-year-old man was admitted to the emergency department for a witnessed cardiac arrest. He had developed an upper respiratory tract infection the preceding week and was prescribed 100 mg of amantadine. Within half an hour of taking the first dose, the patient collapsed. He was found to be in asystole by emergency medical services, and advanced cardiac life support protocols were initiated, including cardiopulmonary resuscitation and intubation for airway protection. However, he sustained multiple recurrences of cardiac arrest, and despite all resuscitation efforts, the patient expired.

## Introduction

Amantadine is a synthetic tricyclic amine derived from adamantane. It was initially indicated as a treatment against the influenza A virus as it inhibits the viral M2 protein, which is a transmembrane protein necessary for viral replication [1,2]. However, it is no longer recommended for use against influenza A as this virus has developed resistance to adamantanes. Currently, amantadine is used to treat Parkinson's disease and drug-induced extrapyramidal symptoms [3]. Although the exact mechanism is not fully understood, it has been reported that amantadine acts on dopaminergic neurotransmission by increasing striatal dopamine release and preventing dopamine reuptake in the presynaptic nerve terminal [2].

Nausea, dizziness/lightheadedness, and insomnia are among the most common adverse effects of amantadine. Other complications include orthostatic hypotension, livedo reticularis, and peripheral edema [4]. In large doses (600 mg to 12 g), amantadine has been reported to cause various types of cardiac arrhythmias [5]. Here, we report a patient who suffered a cardiac arrest after ingesting a starting dose of amantadine.

## Case presentation

A 71-year-old man with a past medical history of hypertension, type 2 diabetes mellitus, hyperlipidemia, and hypothyroidism was brought to the emergency department (ED) for a witnessed cardiac arrest. He developed symptoms of upper respiratory tract infection the preceding week and was prescribed amantadine 100 mg to be taken twice daily for 10 days. Within half an hour of taking the first dose at home, he collapsed. The patient's wife heard a thump and found him unresponsive on the bathroom floor. Emergency medical services (EMS) was called to the scene immediately. In the meantime, she performed cardiopulmonary resuscitation (CPR) until EMS arrived 10 minutes later. The first responders found him to be in asystole. Advanced cardiac life support (ACLS) measures were initiated, and he was transported to the hospital. ACLS was maintained for 10-12 minutes with three rounds of epinephrine.

**Figure 1:**
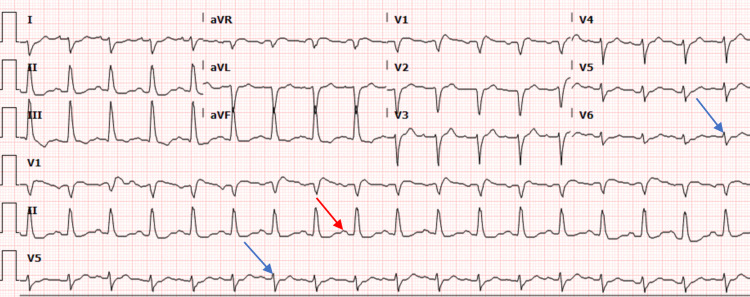
The 12-lead ECG showing sinus tachycardia, nonspecific intraventricular conduction delay (blue arrows), left atrial enlargement (red arrow), and probable anteroseptal infarct

Upon arrival to the ED, the patient was again noted to be in asystole. CPR was resumed per ACLS protocol. Physical examination showed an obese male with a supraglottic airway in place with no signs of chest rise. His pupils measured 4 mm and were fixed bilaterally. No obvious trauma to the face or head was found. The patient was immediately intubated for airway protection and started on invasive mechanical ventilation. After continuous CPR and three rounds of epinephrine, and approximately eight minutes after arrival to the ED, the rhythm converted to sinus tachycardia. His vitals at this time were as follows: blood pressure, 227/101 mmHg; mean arterial pressure, 155 mmHg; heart rate, 115 beats/min (bpm); respiratory rate, 38 breaths/min; temperature, 98.8°F; and oxygen saturation (SpO2), 91% on 100% fraction of inspired oxygen. An electrocardiogram (ECG) revealed sinus tachycardia of 108 bpm, QRS of 131 milliseconds (msec), QTc of 413 msec along with signs of nonspecific intraventricular conduction delay, probable anteroseptal infarct, and left atrial enlargement (Figure 1). He received an ampule of sodium bicarbonate, and subsequent arterial blood gas results showed the following: pH: 6.640; pCO2: 90.9 mmHg; pO2: 91 mmHg; bicarbonate: 9.8 mmol/L; and SpO2: 76%.

The results of point-of-care testing revealed elevated levels of hemoglobin, hematocrit, potassium, blood glucose, and lactate and a low bicarbonate level (Table 1).

**Table 1:** Results of point-of-care testing

Parameter	Reference values	Results
Hemoglobin	14–18 g/dL	21.1
Hematocrit	42–52%	62
Potassium	3.4–4.9 mmol/L	5.4
Bicarbonate	20–29 mEq/L	15
Glucose	70–118 mg/dL	263
Lactate	0.5–2.2 mmol/L	>20.0

Additional laboratory findings are shown in Table 2.

**Table 2:** Laboratory findings WBC - white blood cell; PT - prothrombin time; PTT - partial thromboplastin time; BUN - blood urea nitrogen; eGFR - estimated glomerular filtration rate; AST - aspartate aminotransferase; ALT - alanine aminotransferase; BNP - brain natriuretic peptide

Parameter	Reference value	Results
WBC	4.8–10.8 x10^^3^ /mcL	13.3
PT	12.2–14.9 seconds	20
PTT	23.2–37.4 seconds	66.7
BUN	7–20 mg/dL	18
Creatinine	0.6–1.2 mg/dL	1.5
eGFR	≥60 mL/min/1.73 m^2^	45.43
Anion gap	5–12	32
AST	3–38 unit/L	207
ALT	≤49 unit/L	108
BNP	10–100 pg/mL	128.1

**Figure 2:**
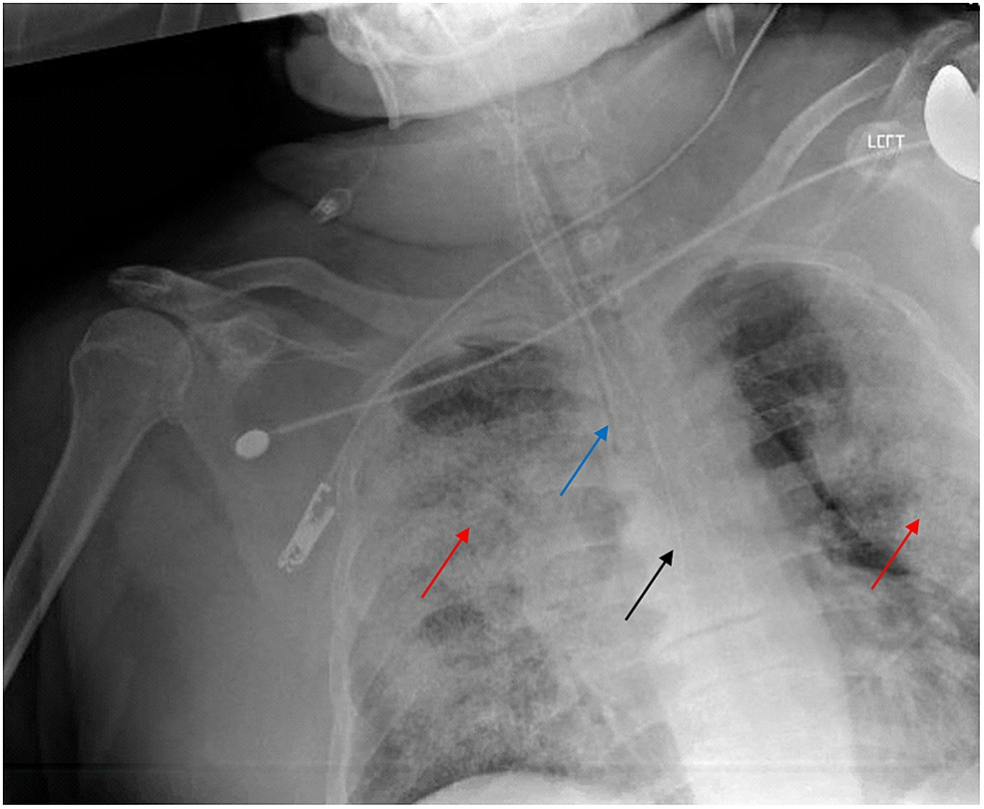
Chest radiograph Chest radiograph showing diffuse bilateral alveolar infiltrates (red arrows). An endotracheal tube terminates above the carina (blue arrow). An orogastric tube extends toward the stomach reaching at least the distal esophagus (black arrow).

Polymerase chain reaction (PCR) for severe acute respiratory syndrome coronavirus 2 (SARS-CoV-2) performed at the ED was positive, whereas those for influenza A/B and respiratory syncytial virus PCR were negative. The chest radiograph showed diffuse bilateral alveolar infiltrates (Figure 2).

At 28 minutes post-presentation to the ED, sinus bradycardia of 55 bpm developed. Atropine and another ampule of sodium bicarbonate were administered. After four minutes, the patient once again appeared to be in asystole. ACLS measures were resumed, and after a round of epinephrine, the rhythm showed sinus tachycardia of 103 bpm, albeit briefly. Approximately 17 minutes later, the patient reverted to asystole, and despite the resumption of CPR and an additional round of epinephrine, no return of spontaneous circulation was achieved. He was subsequently pronounced dead.

## Discussion

The current pandemic has prompted researchers to discover potential therapies for coronavirus disease 2019 (COVID-19), including investigating available drugs indicated for other medical conditions and repurposing them as a treatment for COVID-19. Due to its antiviral effects, amantadine has been suggested as an additional agent that can benefit patients infected with COVID-19 [6]. Amantadine was used as a prophylactic for COVID-19 and as a treatment for patients with COVID-19 in several clinical studies [7-9]. In the study done by Aranda-Abreu et al., 15 patients with clinical presentations of COVID-19 were given amantadine 100 mg twice a day and followed for 14 days [7]. After two weeks of therapy, the patients produced IgG antibodies against the virus and recovered without needing hospitalization or mechanical ventilation. As primary prevention, amantadine was also given to the patients' family members, and none of them developed symptoms of COVID-19. A similar investigation was carried out in patients with Parkinson's disease who were already taking amantadine and were diagnosed with COVID-19 [8]. These patients were quarantined for two weeks; during that period, they showed no symptoms of viral infection.

There are a few possible mechanisms as to how amantadine reduces symptoms caused by COVID-19 [10-13]. It is hypothesized that amantadine blocks the viroporin channel of SARS-CoV-2, thus disrupting the entry of the virus into the cell [10]. Another study based on a gene expression analysis showed that amantadine can downregulate the expression of the cathepsin L gene and lysosomal enzymes, subsequently decreasing viral replication [11]. In conjunction with the proposed mechanisms, a recent in vitro study done in SARS-CoV-2 infected vero E6 cells demonstrated the ability of amantadine to inhibit viral replication [12].

While these findings may favor amantadine as a treatment for COVID-19, further studies are necessary to determine its safety and effectiveness. Amantadine is a drug that is easily absorbed orally. In adults, the daily dose of amantadine is 200 mg. It can be divided into two capsules of 100 mg each. Depending on the indication and patient's response to the drug, the dose may be increased to 400 mg/day. It is important to note that maximum plasma concentrations are strongly associated with dose, and a daily dose of amantadine exceeding 200 mg can lead to a significantly higher increase in maximum plasma concentrations, increasing its toxicity [4]. To prevent and treat the influenza A virus, the daily dose of amantadine for people aged ≥65 years is 100 mg, and similar to drugs with a renal route of excretion, amantadine should be used with caution in elderly patients as well as in individuals with renal insufficiency [3,4]. Previous studies have shown that the pharmacokinetic characteristics of amantadine after administration differ between the young and the elderly [4,14]. After taking a single 100 mg amantadine capsule, the pharmacokinetics of amantadine were measured in 24 healthy adult male volunteers. Time for the maximum concentration (Tmax) ranged between 1.5 to eight hours (h), and the average plasma half-life (T_1/2_) was 17 ± 4 h (range: 10-25 h). However, the average T_1/2_ of amantadine in seven healthy senior male volunteers following a single dose of 25-75 mg administration was 29 ± 7 h (range: 20-41 h) [4]. A comparative study conducted by Hayden et al. in young and elderly adults demonstrated that the elderly had 1.5 times higher peak plasma concentrations and 1.7 times larger area under the curve values than young people after receiving single 200 mg doses of amantadine. In this study, for the elderly adults, Tmax ranged from 0.8 h to 6.4 h, whereas in young adults, Tmax was from 1 h to 3.2 h [14]. In patients older than 60 years old, it was found that the apparent oral plasma clearance of amantadine decreased, leading to increased plasma half-life and concentration of amantadine [4]. The plasma concentration of amantadine was not obtained in this case; however, we believe that the patient experienced toxicity from the accumulation of amantadine due to age-associated reduction in plasma clearance.

Another point to highlight is that the patient described in this case suffered cardiac arrest resulting in death after ingesting a low dose of amantadine. There has only been one case of cardiac arrhythmia caused by 200 mg of amantadine in which the patient developed right ventricular outflow tract tachycardia. In this case, the authors attributed the tachycardia to amantadine and its possible effects on the ventricular muscle and sympathetic nervous system [15]. Previously reported cases of cardiac arrhythmias induced by amantadine were all attributed to high doses, with the lowest and highest doses being 600 mg and 12 g, respectively. In addition, the cardiac arrhythmias described included sinus tachycardia, torsades de pointes, right bundle branch block, sinus bradycardia, pulseless electrical activity, pulseless ventricular tachycardia, and premature ventricular complexes [5]. Deaths from amantadine have also been documented; however, the lowest dose in these cases was 1 g [4]. The exact mechanism of amantadine-induced cardiac arrhythmia has not been established. Cao X et al. have proposed that amantadine may block Na+ and K+ channels and induce Ca2+ channels in the heart in vivo. In addition, proarrhythmic properties of amantadine may be between E-4031 (a human ether-a-go-go related gene potassium channel blocker) and amiodarone [16]. Another study by Hiraoka et al. also reported that the disruption of ionic exchanges can be considered a potential mechanism; however, this was specific for in vitro condition [17].

Cardiac arrest and arrhythmias have also been associated with COVID-19 [18]. Although the exact pathophysiologic mechanisms remain unclear, direct viral infection, hypoxia, electrolyte disturbances, and cytokine storm have been described to cause myocardial injury and increase the risk of arrhythmia in patients with COVID-19 [19-20]. We considered the age-related decline in plasma clearance, the potential proarrhythmic effect of amantadine, and increased risk of arrhythmia due to COVID-19 as factors contributing to the cardiac event described in this case.

## Conclusions

In conclusion, caution should be observed when prescribing amantadine to elderly patients and those with impaired renal function. At present, its use should remain restricted beyond clinical trials as per official guidance from national governing bodies (i.e., the National Institutes of Health). Further comprehensive studies are necessary to assess the safety and effectiveness of amantadine for use in patients with COVID-19. In addition, clinicians need to consider the proarrhythmic effect of COVID-19, particularly when planning to prescribe proarrhythmic medications.
